# The influence of different physical exercise amounts on learning burnout in adolescents: The mediating effect of self-efficacy

**DOI:** 10.3389/fpsyg.2023.1089570

**Published:** 2023-02-20

**Authors:** Wensheng Fu, Yan Li, Yajun Liu, Dan Li, Gang Wang, Yongsen Liu, Tingran Zhang, Yunfeng Zheng

**Affiliations:** ^1^College of General Education, Chongqing Business Vocational College, Chongqing, China; ^2^Sports Work Department, College of Liberal Studies, Chongqing Industry Polytechnic College, Chongqing, China; ^3^Chongqing Xiejiawan School, Chongqing, China; ^4^Chongqing Science City Bashu Secondary School, Chongqing, China; ^5^Department of Physical Education, Xinyang Normal University, Xinyang, China; ^6^Research Centre for Exercise Detoxification, College of Physical Education, Southwest University, Chongqing, China; ^7^Physical Education, Department of Education, School of International Studies, Krirk University, Bangkok, Thailand; ^8^College of Physical Education, Chongqing University of Arts and Sciences, Chongqing, China

**Keywords:** adolescents, physical exercise, self-efficacy, learning burnout, the mediation effect

## Abstract

**Objective:**

To explore the effect of physical exercise on learning burnout in adolescents, and to reveal the mediating effect of self-efficacy between different physical exercise amounts and learning burnout.

**Methods:**

A total of 610 adolescents from 5 primary and middle schools in Chongqing, China were investigated with the Physical Exercise Rating Scale (PARS-3), the General Self-Efficacy Scale (GSES), and the Learning Burnout Scale (LBS). The SPSS21.0 and AMOS21.0 statistical software were used to process and analyze the data.

**Results:**

(1) The physical exercise amount in boys was significantly higher than that in girls, but there was no significant gender difference in self-efficacy and learning burnout. Meanwhile, the academic alienation and low sense of achievement of primary school students were significantly lower than that of junior high school students, and there was no significant difference in the physical exercise amount and self-efficacy. (2) The physical exercise amount in adolescents was positively correlated with self-efficacy (*r* = 0.41), negatively correlated with learning burnout (*r* = −0.46), and self-efficacy was negatively correlated with learning burnout (*r* = −0.45). (3) The physical exercise amount could directly and negatively predict the learning burnout of adolescents (*β* = −0.40), and self-efficacy played a partial mediating effect between the amount of physical exercise and learning burnout (ES = -0.19). (4) Self-efficacy had no significant mediating effect between low exercise amount and learning burnout, but had a significant partial mediating effect between moderate (ES = -0.15) and high exercise amount (ES = -0.22) and learning burnout, and the partial mediating effect between high exercise amount and learning burnout was the highest.

**Conclusion:**

Physical exercise was an effective way to prevent or reduce learning burnout in adolescents. It can not only directly affect learning burnout, but also indirectly affect learning burnout through the mediating effect of self-efficacy. It should be pointed out that maintaining a sufficient amount of physical exercise is crucial to improving self-efficacy and reducing learning burnout.

## 1. Introduction

As a critical stage in life, adolescence is the transitional period from childhood to adulthood, when health-related psychology and behaviors often begin to develop or be reinforced. At this stage, learning was the primary task of teenagers. To cope with the pressure of learning and stand out in the fierce competition, they have to put a lot of energy into learning, which was easy to make teenagers physical and mental fatigue, lack of sense of achievement experience, and easy to hold a negative attitude towards school (Wu et al., 2010) and form learning burnout. Learning burnout refers to psychological factors such as academic pressure during the learning process, resulting in emotional and physical exhaustion, academic alienation, deindividualization, and a low sense of achievement and personal efficacy (Wu et al., 2010; Tuominen-Soini and Salmela-Aro, 2014; Aguayo et al., 2019), and in turn, withdrawal behaviors such as isolation, indifference, and even being late for school and leaving early appear (Li et al., 2019). Studies have shown that learning burnout was significantly related to adolescents’ academic performance (Supervia et al., 2020), and it will not only reduce students’ academic performance and increase the incidence of truancy (Bask and Salmela-Aro, 2013), but also bring negative effects on individual physiology, psychology, behavior, and interpersonal communication, and even lead to suicide behaviors (Wang et al., 2015; Wu J. et al., 2022). Therefore, it is critical to prevent or improve learning burnout in adolescents.

Research in exercise psychology suggested that physical exercise was a personal resource that individuals could use to reduce stress and prevent burnout symptoms (Heuse et al., 2020). There was a strong relationship between physical exercise and learning burnout, as one systematic review suggested that exercise may be the most effective of all interventions to treat learning burnout (Tang et al., 2021). Studies have shown that regular physical exercise has a significant negative correlation with learning burnout, which shows that physical exercise could enhance the mental health of individuals, reduce fatigue and psychological pressure and relieve their tension, and was conducive to the recovery of emotional resources, thus reducing learning burnout (Zhang, 2011; Li et al., 2021). Meanwhile, physical exercise increases heart rate, blood pressure, and the availability of neurotransmitters in the central nervous system, and could also prevent learning burnout by reducing an individual’s stress (Armon, 2014; Khosravi, 2021). This suggested that physical exercise may play a crucial role in reducing learning burnout through psychological and physiological pathways. In college students, physical exercise has also been proven to be effective in reducing learning burnout of college students (Zhou, 2011), however, there were still relatively few studies on adolescents, and the role of physical exercise in reducing learning burnout of adolescents and whether it involves other psychological factors remain unclear.

A study on burnout showed that self-efficacy, as an internal individual characteristic factor, had a great impact on learning burnout (Chen et al., 2016). Self-efficacy refers to how confident people were that they could use their skills to do a certain job, it was often used to explain the reasons for motivation in a special situation, could predict and explain the corresponding behavior, and was the psychological motivation for individual self-regulation to continue (Bandura, 1997). A study has shown that low self-efficacy was one of the important reasons for students’ learning burnout (Yang and Farn, 2005). Students with low self-efficacy have low self-confidence, which will lead to greater learning pressure and anxiety, and they are unable to implement learning behaviors with confidence and obtain a high sense of accomplishment from learning, but feel bored, depressed, or frustrated (Ma et al., 2014). On the contrary, individuals with a higher sense of self-efficacy are more likely to adopt a positive coping style when solving problems, and this behavior could be projected to academic problems, which could arouse the internal learning motivation of individuals (Liem et al., 2008). Interestingly, there was a significant positive correlation between physical exercise amount and adolescents’ self-efficacy (Sheng et al., 2016), and a consistent finding was also found among college students (Hou and Yang, 2016). Studies have shown that physical exercise could effectively improve individuals’ self-efficacy, and it will change adaptively with the depth of exercise (Mcauley et al., 2006; Downs and Strachan, 2016). Individuals’ participation in physical exercise could improve their willpower and endurance, make them feel the pleasure of success, and thus improve their self-efficacy and mental health (Liu, 2020). Further research found that students who actively participated in physical exercise would have relatively higher confidence in their learning ability, thus further promoting their academic performance (Fu and Fan, 2016; Luo et al., 2017). This may be related to the fact that physical exercise can relieve learning fatigue and psychological pressure, thereby relieving bad moods and reducing learning burnout (Xue et al., 2022). In this process, based on ensuring the necessary time and energy invested in learning, self-efficacy may play a unique role, as Sheng et al. (2016) pointed out that self-efficacy plays a mediating role between physical exercise and the mental health of adolescents. So, does self-efficacy also play a mediating role between physical exercise and learning burnout in adolescents? In addition, physical exercise amount plays a significant role in enhancing the learning emotion and self-efficacy of high school students, and there was a dose effect of exercise amount on self-efficacy (Zhao et al., 2019). It was shown that the greater the amount of physical exercise, the lower the students’ academic fatigue, and the stronger their self-confidence and sense of achievement, which is more conducive to eliminating or reducing psychological barriers such as learning burnout (Zhang, 2011). The results suggested that the effect of physical exercise on individual mental health may have a dose effect.

To sum up, heavy academic pressure and negative emotions tend to lead to learning burnout among adolescents, while physical exercise and self-efficacy may be important variables affecting learning burnout in adolescents. However, there were few previous studies on the relationship between physical exercise, self-efficacy, and learning burnout in adolescents, and whether there was a dose-effect of exercise amount in the path of physical exercise affecting learning burnout was rarely. Based on this, this study puts forward the following hypotheses: (H1) Physical exercise has a direct negative predictive effect on learning burnout of adolescents. (H2) Self-efficacy has a direct negative predictive effect on the learning burnout of adolescents. (H3) Self-efficacy has a mediating effect between physical exercise and learning burnout. (H4) The influence of different exercise amounts on learning burnout was different. By verifying the research hypothesis, this study explored the influence of physical exercise on the learning burnout of adolescents and revealed the mediating effect of self-efficacy between different physical exercise amounts and learning burnout.

## 2. Materials and methods

### 2.1. Participants

This study adopted a cross-sectional research design and, conducted questionnaires among students at two primary schools and three junior high schools in Chongqing, China, from April to June 2022. Among them, students in two primary schools need to spend about 9 h a day in school (Starting at 8 AM and finishing at 5 PM), with eight classes a day. Physical activities are composed of informal (Free activities between classes) and formal (Morning exercise and physical education classes), and three classes of physical education classes every week (Monday, Wednesday, and Friday). These students in three junior high schools need to spend about 9.5 h a day in school (Starting at 8 AM and finishing at 5.30 PM), with nine classes a day. Physical activities are composed of informal (Free activities between classes) and formal (Morning exercise and physical education classes), and three classes of physical education classes every week (Monday, Tuesday, and Thursday).

According to the actual number of students in each school, random sampling was conducted with student numbers at a ratio of about 1:100, and 120−150 people were stratified from each school to conduct a questionnaire survey (including elementary grades 4 to 6 and junior high school grade 1 to 3). Inclusion criteria: (1) Age 9 to 15 years old; (2) Full-time students; (3) Can carry out normal physical activity or physical exercise; (4) Volunteer to participate in this study. Exclusion criteria: (1) People with intellectual disabilities; (2) People with mental disorders. The selected students were told to participate in the study and were sent to a designated classroom to fill out the questionnaire and collect the questionnaire on-site. The participants needed to spend about 16 min in class to complete the questionnaire. Two weeks before the formal test, to verify the reliability of the questionnaire, 15% of the total samples were randomly selected for re-test. After filling in the questionnaire in the first week, these participants filled in the questionnaire again 1 week later. After passing the re-test (104 students), a formal questionnaire survey was conducted on all the samples, these students were included in the main part of the study. A total of 698 questionnaires were distributed and 677 were recovered, with a recovery rate of 96.99%. After eliminating invalid questionnaires with unknown key information and incomplete questionnaires, 610 valid questionnaires were finally obtained, with an effective rate of 90.10%. The average age of the participants was 13.79 ± 3.25 years, and 363 boys (59.51%) and 247 girls (40.49%). There were 231 students (37.87%) in primary schools, among them, 62 students (10.16%) were in the fourth grade, 76 students (12.46%) were in the fifth grade, and 93 students (15.25%) were in the sixth grade. And 379 students (62.13%) in junior high schools, among them, 145 (23.77%) in the first grade of middle school, 128 (20.98%) in the second grade of middle school, and 106 (17.38%) in the third grade of middle school. The study was approved by the Ethics Committee of Southwest University Hospital (202105), and written informed consent was obtained from all participants in compliance with the Declaration of Helsinki. Meanwhile, due to the survey subjects being minors, we also obtained the approval of the participants’ parents in advance and signed the informed consent of the parents.

### 2.2. Measurement tools

#### 2.2.1. Physical exercise rating scale (PARS-3)

The Physical Activity Rating Scale of Liang (1994) was used and revised to evaluate the physical exercise of participants from three aspects: exercise intensity, frequency, and time. The Likert 5-point scoring method was used to quantify the items, among them, the exercise intensity and frequency from weak to strong were, respectively, calculated as 1 to 5 points, and exercise time from weak to strong was, respectively, calculated as 0 to 4 points. The formula “exercise intensity × exercise time × exercise frequency” was used to quantify the total score of exercise behavior, with the score ranging from 0 to 100, and the higher the score, the greater the exercise amount. Meanwhile, the assessment standard of physical activity was: low exercise amount of ≤19 points, moderate exercise amount of 20–42 points, and high exercise amount of ≥43 points. The re-test showed that the load of each item of the PARS-3 was between 0.50 and 0.95, the combined reliability (CR) was greater than 0.6, and the average variance extraction value (AVE) was also greater than 0.5, indicating that the convergent validity of the scale was good. Moreover, the AVE value was greater than the square value of the correlation coefficient, indicating good discriminant validity. After factor analysis of the scale, a common factor was extracted, and the common factor contained 10 items, and the progressive contribution rate of the common factor was 56.19%. Through the internal consistency test, the overall Cronbach α coefficient of the scale was 0.82. The confirmatory factor analysis results were as follows: *x*^2^/df = 2.13, RMSEA = 0.06, AGFI = 0.95, TLI = 0.98, CFI = 0.96, IFI = 0.96, GFI = 0.97. The results indicated that the scale had good reliability and validity.

#### 2.2.2. General self-efficacy scale

The General Self-efficacy Scale, revised by Wang et al. (2001), contains 10 questions and belongs to the single-dimensional structure scale. The Likert 4-point scale was used for quantification, and according to the options “disagree ~ strongly agree,” the score ranged from 1 to 4, and the score range was from 10 to 40. The higher the score, the stronger the sense of self-efficacy perceived by the individuals. The re-test showed that the load of each item of the GSES was between 0.50 and 0.95, the combined reliability (CR) was greater than 0.6, and the average variance extraction value (AVE) was also greater than 0.5, indicating that the convergent validity of the scale was good. Moreover, the AVE value was greater than the square value of the correlation coefficient, indicating good discriminant validity. After factor analysis of the scale, a common factor was extracted, and the common factor contained 10 items, and the progressive contribution rate of the common factor was 57.68%. Through the internal consistency test, the overall Cronbach α coefficient of the scale was 0.86. The confirmatory factor analysis results were as follows: *x*^2^/df = 1.95, RMSEA = 0.05, AGFI = 0.97, TLI = 0.98, CFI = 0.95, IFI = 0.95, GFI = 0.98. The results indicated that the scale had good reliability and validity.

#### 2.2.3. Learning burnout scale

The Learning Burnout Scale was created by Wu et al. (2010). The scale has a total of 16 items, which were quantified by the Likert 5-point score, ranging from 16 to 80 points. The higher the score, the deeper the degree of learning burnout perceived by the individuals. The re-test showed that a load of each item of the LBS was between 0.50 and 0.95, the combined reliability (CR) was greater than 0.6, and the average variance extraction value (AVE) was also greater than 0.5, indicating that the convergent validity of the scale was good. Moreover, the AVE value was greater than the square value of the correlation coefficient, indicating good discriminant validity. A total of 3 common factors were extracted after factor analysis of the scale. After direct oblique rotation, the 3 common factors contained 16 items, and the progressive contribution rate of the 3 common factors reached 61.02%. According to the internal consistency test, the Cronbach α coefficients of the three dimensions, namely physical and mental exhaustion (4 items), academic alienation (5 items), and low sense of achievement (7 items), were 0.87, 0.88, and 0.82, respectively. The confirmatory factor analysis results were as follows: *x*^2^/df = 2.03, RMSEA = 0.06, AGFI = 0.98, TLI = 0.93, CFI = 0.97, IFI = 0.95, GFI = 0.98. The results indicated that the scale had good reliability and validity. The details of the three scales were shown in Table 1.

**Table 1:** Factor extraction and reliability analysis of the three measurement scales.

Scales	KMO and Bartlett ball detection	Dimension	Items	Characteristics of the root	Explained variation (%)	Progressive explained variation (%)	Cronbach’s α coefficient
PARS-3	KMO = 0.87	Physical exercise amount	3	4.17	55.45	55.45	0.82
	(*p* < 0.001)						
LBS	KMO = 0.89	Exhaustion of body and mind	4	6.19	31.38	31.38	0.87
	(*p* < 0.001)	Academic alienation	5	4.55	19.46	50.84	0.88
		Low sense of achievement	7	2.47	10.18	61.02	0.82
GSES	KMO = 0.91	Self-efficacy	10	5.43	57.68	57.68	0.86
	(*p* < 0.001)						

### 2.3. Data analysis

SPSS21.0 was used to process and analyze the data. Quantitative variables were mainly tested by parameters, which mainly include the following: The factor analysis and internal consistency tests were used to investigate the reliability and validity of the scale used for quantitative variables. The descriptive statistical analysis and independent sample t-test were used to investigate the demographic differences in physical exercise, self-efficacy, and learning burnout among adolescents. Pearson correlation was used to analyze the correlation between variables, regression analysis was used to explore the linear relationship between variables, AMOS 21.0 was used to build a structural equation model to reveal the mediating effect of self-efficacy, and Bootstrap analysis was used to test the mediating effect. The significance level of all indexes was set at *p* < 0.05.

## 3. Results

### 3.1. Demographic difference analysis

Through demographic difference analysis found that (Table 2): (1) In terms of gender, the MPEA of boys was significantly higher than that of girls (*t* = 4.04, *p* < 0.05), but there was no significant gender difference between LPEA (*t* = 1.21, *p* > 0.05) and HPEA (*t* = 1.30, *p* > 0.05). Meanwhile, there were no significant gender differences in SE (*t* = 1.06, *p* > 0.05), PME (*t* = 0.89, *p* > 0.05), AA (*t* = 1.04, p > 0.05), and LSA (*t* = 1.10, *p* > 0.05). (2) In terms of academic stage, AA of primary school students (*t* = 2.98, *p* < 0.05) and LSA (*t* = 3.76, *p* < 0.05) were significantly lower than those of junior middle school students, and there was no significant difference in SE (*t* = 2.30, *p* > 0.05) and PME (*t* = 0.90, *p* > 0.05).

**Table 2:** Differences in the physical exercise amount, self-efficacy, and learning burnout among adolescents.

Variable	Physical exercise amount			SE	Learning burnout		
	LPEA	MPEA	HPEA		PME	AA	LSA
Boys	18.03 ± 4.26	31.15 ± 5.30	45.02 ± 6.78	25.32 ± 4.01	15.56 ± 3.48	23.47 ± 4.19	33.77 ± 5.79
Girls	16.95 ± 4.08	26.39 ± 4.87	43.11 ± 6.35	24.08 ± 4.12	15.02 ± 3.30	22.19 ± 4.03	32.08 ± 5.27
*t*	1.21	4.04*	1.30	1.06	0.89	1.04	1.10
*p*	0.45	0.02	0.41	0.59	0.65	0.56	0.50
Primary school	17.78 ± 4.18	30.67 ± 5.03	44.13 ± 6.66	23.64 ± 3.91	15.44 ± 3.31	22.58 ± 4.06	31.68 ± 5.22
Junior middle school	17.02 ± 4.09	28.33 ± 4.96	43.17 ± 6.09	25.80 ± 4.19	15.96 ± 3.43	25.11 ± 4.62	35.10 ± 5.79
*t*	0.68	1.34	1.15	2.30	0.90	2.98*	3.76*
*p*	0.71	0.37	0.49	0.09	0.60	0.04	0.03

^*^Indicates *p* < 0.05. LPEA indicates low physical exercise amount; MPEA indicates moderate physical exercise amount; HPEA indicates high physical exercise amount; SE indicates self-efficacy; PME indicates physical and mental exhaustion; AA indicates academic alienation, and LSA indicates a low sense of achievement.

### 3.2. Correlation analysis between variables

The Pearson correlation analysis showed (Table 3) that the physical exercise amount of adolescents was positively correlated with self-efficacy (*r* = 0.41, *p* < 0.01), and with learning burnout (*r* = −0.46, *p* < 0.001), physical and mental exhaustion (*r* = −0.31, *p* < 0.05), academic alienation (*r* = −0.40, *p* < 0.01), and low sense of achievement (*r* = −0.48, *p* < 0.001) were significantly negatively correlated, respectively. Self-efficacy was negatively correlated with learning burnout (*r* = −0.45, *p* < 0.001), physical and mental exhaustion (*r* = −0.37, *p* < 0.01), academic alienation (*r* = −0.36, *p* < 0.01), and low sense of achievement (*r* = −0.41, *p* < 0.01), respectively. It can be seen that the correlation between each main variable reaches the significance level, which provides a good foundation for the subsequent test of mediating effect.

**Table 3:** Correlation between physical exercise amount, self-efficacy, and learning burnout.

Variable	M ± SD	PEA	SE	LB	PME	AA	LSA
PEA	29.46 ± 5.28	1	1	1	1	1	1
SE	24.71 ± 4.06	0.41^**^					
LB	50.32 ± 6.99	−0.46^***^	−0.45^***^				
PME	10.17 ± 3.11	−0.31^*^	−0.37^**^	0.82^***^			
AA	15.67 ± 3.86	−0.40^**^	−0.36^**^	0.87^***^	0.51^***^		
LSA	24.50 ± 5.07	−0.48^***^	−0.41^**^	0.75^***^	0.60^***^	0.55^***^	

^*^p < 0.05, ^**^p < 0.01, ^***^p < 0.001. PEA indicates physical exercise amount; LB indicates learning burnout.

### 3.3. The influence path of physical exercise on learning burnout

#### 3.3.1. Direct effect analysis

The Linear regression analysis was used to test the direct relationship among variables (Table 4). Firstly, after controlling for demographic variables such as gender and age, this study took the physical exercise amount as the independent variable, and self-efficacy and learning burnout as the dependent variable, respectively. The results showed that the physical exercise amount could positively predict the self-efficacy of adolescents (*β* = 0.41, *p* < 0.01), which could explain 16% of the variation. Physical exercise also negatively predicted learning burnout (*β* = −0.46, *p* < 0.001), which could explain 21% of the variation. Secondly, taking adolescents’ self-efficacy as the independent variable and learning burnout as the dependent variable, self-efficacy could negatively predict learning burnout (*β* = −0.45, *p* < 0.001), which could explain 20% of the variation.

**Table 4:** The linear regression analysis of physical exercise amount, self-efficacy, and learning burnout.

Variable	Self-efficacy			Learning burnout		
	*β*	*R* ^2^	95%CI	*β*	*R* ^2^	95%CI
Physical exercise amount	0.41^**^	0.17	(0.38,0.44)	−0.46^***^	0.21	(−0.53,-0.41)
Self-efficacy				−0.45^***^	0.20	(−0.50,-0.42)

^**^p < 0.01, ^***^p < 0.001.

#### 3.3.2. Mediating effect analysis

**Figure 1:**
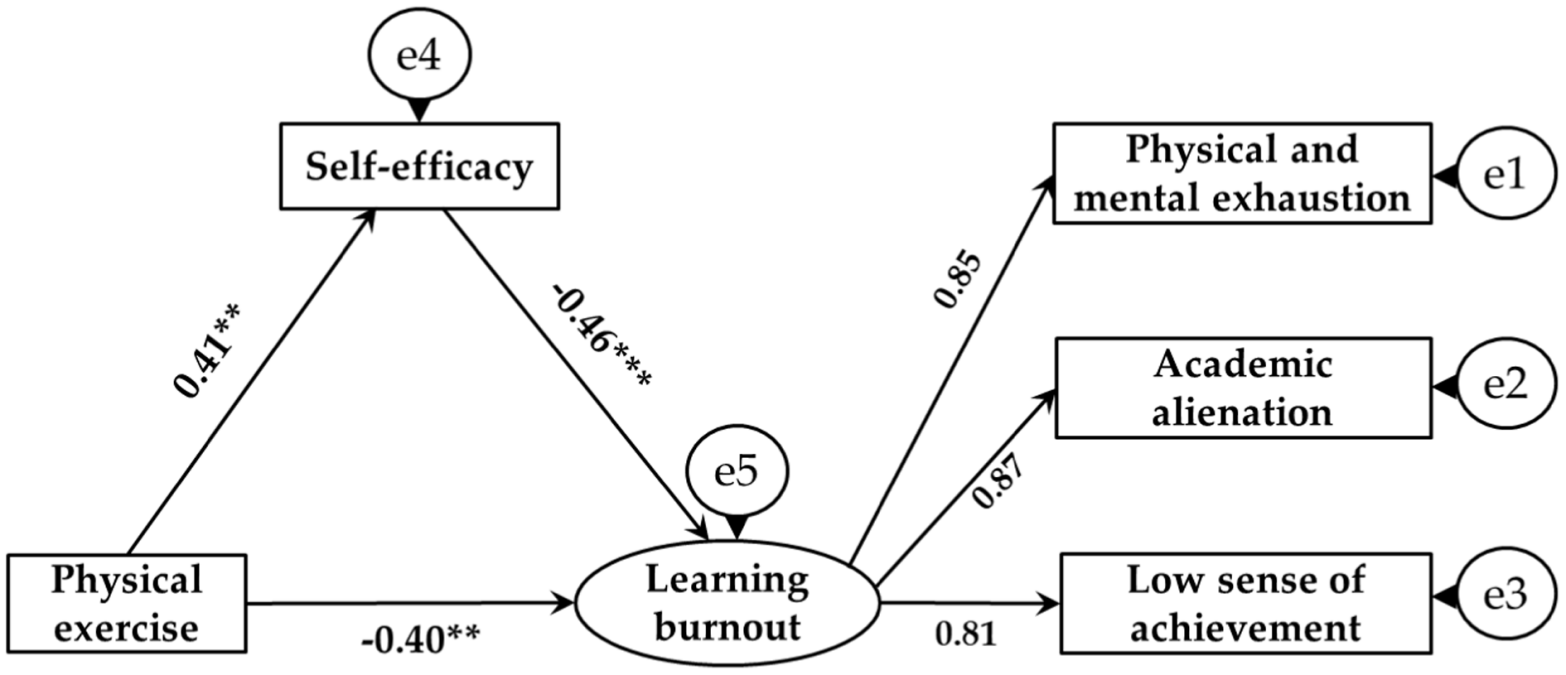
The mediating path model diagram of physical exercise influencing learning burnout. ^**^p < 0.01, ^***^p < 0.001.

In this study, Baron and Kenny’s (1986) mediation test method was used for reference, and AMOS software was used to establish a structural equation model to investigate the mediating effect of self-efficacy on adolescents’ physical exercise and learning burnout. The fitting indexes of the model were as follows: *x*^2^/df = 1.92, RMSEA = 0.04, GFI = 0.95, TLI = 0.98, NFI = 0.94, IFI = 0.92, AGFI = 0.96, the model has a good fit, indicating that it was suitable for mediating effect test. The model results showed (Figure 1) that the path coefficient of physical exercise on learning burnout was significant (*β*1 = −0.46, SE = 0.03, *p* < 0.001). After adding self-efficacy as a mediating variable, the path coefficient of physical exercise on self-efficacy was significant (*β* = 0.41, SE = 0.03, *p* < 0.01), and the path coefficient of self-efficacy on learning burnout was significant (*β* = −0.46, SE = 0.02, *p* < 0.001). However, the path coefficient of physical exercise on learning burnout decreased, but still reached a significant level (*β*2 = −0.40, SE = 0.04, *p* < 0.01), indicating that self-efficacy had a partial mediating effect (see Table 5 for the effect decomposition of each path). Therefore, hypotheses H1, H2, and H3 of this study have been confirmed.

**Table 5:** The list of path effects.

Category	Standardized effect value	The proportion of the total effect	Bootstrap SE	Significant
Total effect	−0.59	100%	0.02	Significant
Direct effect	−0.40	67.80%	0.04	Significant
Indirect effect	−0.46 × 0.41 = −0.19	32.20%	0.02	Significant

To further verify whether self-efficacy has a mediating effect between different amounts of physical exercise and learning burnout, this study adopts SPSS macro Model 4 compiled by Hayes (2012) to estimate the 95% confidence interval of mediating effect by sampling 5,000 samples and carries out mediating effect test in different mediating models. If the 95% confidence interval of the mediating effect does not include 0, the mediating effect was significant, otherwise, the mediating effect was insignificant. The mediating effect test of this study was carried out based on controlling statistical variables such as gender, and academic stage (or age).

Regression analysis results showed that (Tables 6–8): (1) Low exercise amount had no significant predictive effect on self-efficacy (*β* = 0.19, *p* > 0.05), but self-efficacy could significantly negatively predict learning burnout (*β* = −0.25, *p* < 0.05), and when both of them predicted learning burnout, low exercise amount could significantly negatively predict learning burnout (*β* = −0.27, *p* < 0.05). (2) Moderate exercise amount could significantly positively predict self-efficacy (*β* = 0.37, *p* < 0.01), and self-efficacy could significantly negatively predict learning burnout (*β* = −0.40, *p* < 0.01), and when both of them predicted learning burnout, moderate exercise amount could significantly negatively predict learning burnout (*β* = −0.34, *p* < 0.05). (3) High exercise amount could significantly positively predict self-efficacy (*β* = 0.45, *p* < 0.001), and self-efficacy could significantly negatively predict learning burnout (*β* = −0.48, *p* < 0.001), and when both of them predicted learning burnout, high exercise amount could significantly negatively predict learning burnout (*β* = −0.39, *p* < 0.01).

**Table 6:** Linear regression analysis of self-efficacy between low physical exercise amount and learning burnout (*n* = 240).

Variable	Self-efficacy			Learning burnout		
	*β*	*t*	95%CI	*β*	*t*	95%CI
Low physical exercise amount	0.19	1.47	(−0.05,0.23)	−0.27^*^	2.98	(−0.34,-0.21)
Self-efficacy				−0.25^*^	2.79	(−0.29,-0.18)

^*^p < 0.05.

**Table 7:** Linear regression analysis of self-efficacy between moderate physical exercise amount and learning burnout (*n* = 218).

Variable	Self-efficacy			Learning burnout		
	*β*	*t*	95%CI	*β*	*t*	95%CI
Moderate physical exercise amount	0.37^**^	3.88	(0.34,0.42)	−0.34^*^	3.60	(−0.39,-0.31)
Self-efficacy				−0.40^**^	4.03	(−0.46,-0.38)

^*^p < 0.05, ^**^p < 0.01.

**Table 8:** Linear regression analysis of self-efficacy between high physical exercise amount and learning burnout (*n* = 152).

Variable	Self-efficacy			Learning burnout		
	*β*	*t*	95%CI	*β*	*t*	95%CI
High physical exercise amount	0.45^***^	4.92	(0.41,0.53)	−0.39^**^	3.99	(−0.44,-0.35)
Self-efficacy				−0.48^***^	5.11	(−0.51,-0.42)

^**^p < 0.01; ^***^p < 0.001.

**Figure 2:**
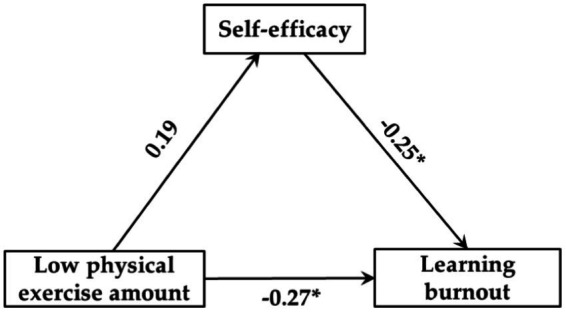
Mediating model of self-efficacy between low exercise amount and learning burnout.

**Figure 3:**
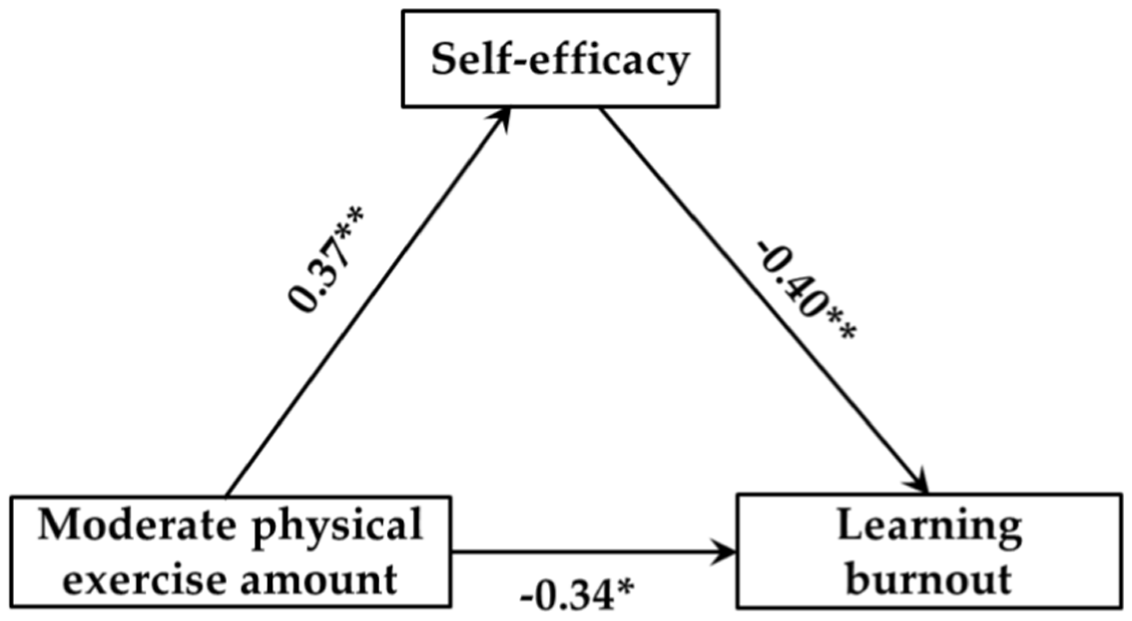
Mediating model of self-efficacy between moderate exercise amount and learning burnout.

**Figure 4:**
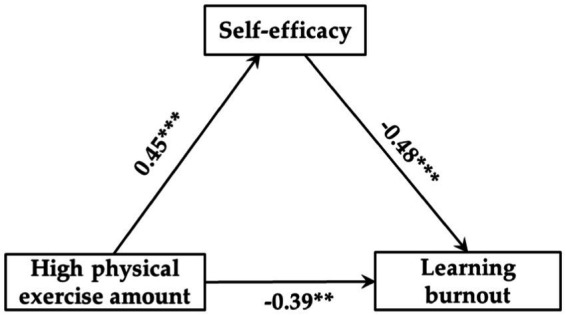
Mediating model of self-efficacy between high exercise amount and learning burnout. ^*^p < 0.05, ^**^p < 0.01, ^***^p < 0.001.

Figures 2–4 showed the mediating effect of self-efficacy between low-, moderate-, and high exercise amount and learning burnout, respectively. The results of this study showed that: (1) “low physical exercise amount → self-efficacy → learning burnout,” the confidence interval of this path contains 0, indicating that self-efficacy does not have a significant mediating effect between low exercise amount and learning burnout, and the direct predictive effect of low exercise amount on self-efficacy was not significant. (2) “moderate physical exercise amount → self-efficacy → learning burnout,” the confidence interval of this path does not contain 0, showing that self-efficacy had a significant mediating effect between moderate exercise amount and learning burnout (the standardized effect value was 0.15, accounting for 30.61% of the total effect), and moderate exercise quantity direct prediction of learning burnout was significant, so the self-efficacy plays a partial mediating role between moderate exercise amount and learning burnout. (3) “High physical exercise amount → self-efficacy → learning burnout,” indicating that self-efficacy had a significant mediating effect between high exercise amount and learning burnout (the standardized effect value was −0.22, accounting for 36.07% of the total effect), and high exercise amount has a significant direct predictive effect on learning burnout, so the self-efficacy plays a partial mediating role between high exercise amount and learning burnout. Therefore, hypothesis H4 of this study has been confirmed.

To sum up, physical exercise can not only directly predict learning burnout in adolescents, but also indirectly predict learning burnout through the partial mediating effect of self-efficacy. Among them, the mediating effect of self-efficacy between low exercise amount and learning burnout was not significant, while the partial mediating effect of self-efficacy between moderate and high exercise amount and learning burnout was significant, and the partial mediating effect between high exercise amount and learning burnout was the highest.

## 4. Discussion

This study found that there were some differences in physical exercise, self-efficacy, and learning burnout among adolescents of different genders and academic stages. Meanwhile, the physical exercise amount could directly negatively predict the learning burnout of adolescents, and self-efficacy plays a partial mediating role between moderate exercise amount and learning burnout as well as between high exercise amount and learning burnout. However, the partial mediating effect of self-efficacy was the highest between the high exercise amount and learning burnout.

### 4.1. Difference analysis

This study found that there was a significant gender difference in the amount of physical exercise among adolescents, which showed that the physical exercise amount of boys was significantly higher than that of girls. Studies have shown that, compared with girls, boys will maintain relatively independent, stable, and regular exercise habits in their spare time due to their stronger exercise motivation and desire (Davies and Lindsay, 2004; Xu and Dong, 2020), and thus were more likely to engage in more exercise behaviors. Meanwhile, in terms of personality traits, boys were usually more active in adolescence (Raudsepp, 2006). In addition, influenced by traditional social consciousness and cultural thoughts, girls were more introverted and spontaneous in exercise, and it was difficult for girls to maintain regular and stable exercise behavior (Dong and Zhang, 2016). However, this study did not find significant gender differences in self-efficacy and learning burnout in adolescents. We speculate that the psychological characteristics of boys and girls in adolescence were in the rapid growth stage, the external environment cognition has not formed significant psychological differences, and the selected samples were all from the same city, which may limit the occurrence of such differences to a certain extent. In addition, for adolescents at different academic stages, this study only found significant differences in learning burnout, while there was no significant difference in the amount of physical exercise and self-efficacy, showing that the learning burnout of primary school students was significantly lower than that of junior high school students. This may be because junior high school students in China usually face more subject classes or homework and were under greater pressure to go to school, while primary school students were more likely to complete basic studies and develop various interests, so learning burnout was lower.

### 4.2. The direct influence of physical exercise on learning burnout of adolescents

When adolescents were under long-term academic pressure and overload, they were prone to a low sense of accomplishment, emotional exhaustion, and learning burnout with bad learning behaviors (Schaufeli et al., 2002), and long-term learning burnout will easily lead to an increase in the incidence of mental health problems in adolescents (Wu J. et al., 2022). A study has shown that active participation in physical exercise was negatively correlated with learning burnout of primary school students, and could negatively predict their learning burnout (Xue et al., 2022). Li et al. (2021) also showed in a study on Chinese adolescents that physical exercise had a negative direct predictive effect on learning burnout, and then effectively improved their psychological distress. Based on previous studies, this study confirmed the negative correlation between physical exercise and learning burnout in adolescents, and the physical exercise amount has a linear relationship with learning burnout and can negatively predict learning burnout. In other words, the higher the physical exercise amount, the lower the learning burnout perceived by the individuals. This can be interpreted as regular physical exercise, as an effective intervention means, can relieve the adverse emotional state by reducing fatigue and psychological pressure, promote the recovery of emotional resources, improve the mental health of individuals, and effectively reduce learning burnout (Zhang, 2011; Tang et al., 2021; Xue et al., 2022). The results suggested that adolescents should develop and maintain the habit of good physical exercise, which was an effective means to prevent learning burnout and other adverse mental health problems. Meanwhile, when adolescents have learning burnout, physical exercise can also effectively improve the degree of learning burnout.

### 4.3. The mediating effect of self-efficacy between physical exercise and learning burnout

This study found that self-efficacy played a significant partially mediating effect in the path of physical exercise negatively affecting learning burnout in adolescents. Firstly, there was a mutually promoting relationship between physical exercise behavior and self-efficacy, which showed that active sports participation could positively affect participants’ self-efficacy, improve their satisfaction with life, and thus promote their physical and mental health development (Wang and Ma, 2014; Downs and Strachan, 2016; Zhao et al., 2019). For example, an early study has found that individuals’ self-efficacy will change positively after strenuous running or cycling (McAuley et al., 1995). A study on adolescents has found that if they take active physical exercise, their self-efficacy, academic mood, and academic performance could be effectively improved, and their level of physical and mental health could be promoted (Zhao et al., 2019). Yuan and Zhang (2015) also found that the physical exercise amount has a significant correlation with adolescents’ self-efficacy and other mental health, and has a good predictive effect on self-efficacy. Among college students, there was a significant positive correlation between physical exercise and self-efficacy, that is, the higher the degree of physical exercise, the higher the self-efficacy of college students (Hou and Yang, 2016; Zhao et al., 2019). Secondly, there was a significant correlation between self-efficacy and learning burnout. A study has shown that the self-efficacy of primary school students was significantly negatively correlated with learning burnout, and self-efficacy has a significant negative predictive effect on learning burnout (Xue et al., 2022). Liao (2010) also pointed out that students’ self-efficacy was significantly negatively correlated with learning burnout, which could be regarded as a predictive variable of learning burnout. According to the research, students with a low sense of self-efficacy often have low self-confidence, which makes it difficult for them to obtain a high sense of accomplishment in the process of learning, and feel bored, depressed, or frustrated, which easily leads to greater learning pressure and anxiety, and eventually leads to learning burnout (Ma et al., 2014). On the contrary, with the improvement of self-efficacy, the expectation of efficacy will also be greatly increased, to awaken the internal learning motivation of individuals, stimulate the desire and pleasure of learning (Duan and Hong, 2019), and thus reduce the burnout of teenagers in learning. These results suggested that self-efficacy may play a unique role in the relationship between physical exercise and learning burnout in adolescents. Meanwhile, self-efficacy has a partial mediating effect between physical exercise and learning burnout, showing that after-school physical exercise could not only directly affect the learning burnout of primary school students, but also indirectly affect learning burnout through cognitive participation and self-efficacy (Xue et al., 2022). It shows that adolescents’ participation in physical exercise amount can also indirectly affect negative emotions through self-efficacy (Wu J. T. et al., 2022), which may further reduce or prevent learning burnout. Interestingly, this study confirmed that self-efficacy has a partial mediating effect between physical exercise amount and learning burnout in adolescents, with a mediating effect size of −0.19. That is, excluding the influence of factors such as gender and academic stage, physical exercise could not only directly negatively predict learning burnout, but also have a negative predictive effect on learning burnout through the partial mediating effect of self-efficacy.

In addition, this study found differences in the partial mediating effect of self-efficacy between different amounts of physical exercise and learning burnout. It showed that self-efficacy had no significant mediating effect between low exercise amount and learning burnout, while it had a significant partial mediating effect between moderate–and high exercise amount and learning burnout, and the partial mediating effect between high exercise amount and learning burnout was the highest. Previous studies have shown that different intensity of exercise has significant differences in individual self-efficacy, and the higher the degree of physical exercise, the higher the self-efficacy perceived by the individuals (Hou and Yang, 2016; Zhao et al., 2019). The self-efficacy will gradually increase with the increase of the exercise time and exercise level of the individual, improving the participants’ satisfaction with life, and thus promoting their physical and mental health development (Wang and Ma, 2014; Downs and Strachan, 2016). The results of this study showed that there was a linear relationship between the amount of physical exercise and the self-efficacy of adolescents, but low exercise amount was difficult to achieve the purpose of improving self-efficacy. Meanwhile, Zhao et al. (2019) further studied and found that different intensities of exercise had different effects on students’ self-efficacy and learning emotion, but there was no significant difference between the low exercise group and the control group, indicating that physical exercise needs to reach a certain level to achieve the purpose of improving academic performance. The greater the physical exercise amount, the more conducive students were to reduce fatigue and enhance self-confidence and sense of achievement, reduce depression, pessimism, and other negative emotions, and then eliminate or reduce learning burnout and other psychological barriers (Zhang, 2011). It can be seen that although the correlation between physical exercise, self-efficacy, and learning burnout was supported by most studies, it was necessary to maintain a moderate or high amount of exercise in adolescents to improve their self-efficacy and effectively prevent or reduce their learning burnout. In this way, it can not only give full play to the direct improvement benefit of physical exercise on learning burnout but also maximize the mediating effect of self-efficacy. We speculate that the intrinsic relationship between physical exercise, self-efficacy, and learning burnout is widely applicable to the majority of healthy adolescents. Therefore, adolescents should participate in physical exercise as much as possible, which is one of the effective ways to promote their mental health.

### 4.4. Limitations

This study mainly discusses the effect of physical exercise on learning burnout in adolescents and reveals the mediating effect of self-efficacy between different amounts of physical exercise and learning burnout, which provides a reference for preventing or alleviating learning burnout caused by learning pressure in adolescents. However, as this study was a cross-sectional study, the results obtained were more subjective and cannot draw a deeper causal relationship. Therefore, the experimental study or clinical study can be added to future studies to better reveal the causal association between variables and clinical evidence. Meanwhile, the sample of this study only involves elementary and junior high school students in Chongqing, China, so the conclusions drawn have certain geographical limitations. Future research can involve a wider range of respondents and conduct a cross-regional comparison. In addition, this study mainly examines the mediating effect of self-efficacy, and specific personality traits (particularly novelty-seeking and cooperativeness personality traits) related to individual physical activity level and academic burnout (Khosravi et al., 2020). Therefore, more mediating or moderating variables can be added in the future to expand the depth and breadth of the study. Finally, when analyzing the relationship between physical exercise and learning burnout, this study did not control and analyze the time adolescents spent on learning or physical activities in school. Therefore, follow-up studies can fully consider these potential confounding factors, to promote a clearer relationship between variables.

## 5. Conclusion

There was a significant correlation between physical exercise, self-efficacy, and learning burnout in adolescents. Among them, the physical exercise amount could directly negatively predict learning burnout, and self-efficacy plays a partial mediating role between the physical exercise amount and learning burnout. After quantitative grading of physical exercise, it was found that the mediating effect of self-efficacy between low exercise amount and learning burnout was not significant, while both moderate–and high exercise amount had significant partial mediating effects on learning burnout, and the partial mediating effect between high exercise and learning burnout was the highest. Teenagers should actively participate in physical exercise and try to maintain a moderate level of exercise, which is more conducive to improving their self-efficacy and preventing or improving learning burnout.

## Data availability statement

The original contributions presented in the study are included in the article/supplementary material, further inquiries can be directed to the corresponding authors.

## Ethics statement

The studies involving human participants were reviewed and approved by the Ethics Committee of Southwest University Hospital. Written informed consent to participate in this study was provided by the participants’ legal guardian/next of kin.

## Author contributions

WF and YnL carried out the protocol and wrote the first draft. YjL carried out the questionnaire survey. WF and DL recruited the participants. YnL and GW undertook the statistical analysis and graphical representation of the data. TZ provides financial support. YoL and ZY revised the draft. All authors designed this study and contributed to the article and approved the final manuscript.

## Funding

This study was supported by the Fundamental Research Funds for the Central Universities (SWU1909438).

## Conflict of interest

The authors declare that the research was conducted in the absence of any commercial or financial relationships that could be construed as a potential conflict of interest.

## Publisher’s note

All claims expressed in this article are solely those of the authors and do not necessarily represent those of their affiliated organizations, or those of the publisher, the editors and the reviewers. Any product that may be evaluated in this article, or claim that may be made by its manufacturer, is not guaranteed or endorsed by the publisher.
